# Regulation of host and viral promoters during human cytomegalovirus latency via US28 and CTCF

**DOI:** 10.1099/jgv.0.001609

**Published:** 2021-05-27

**Authors:** Elizabeth G. Elder, Benjamin A. Krishna, Emma Poole, Marianne Perera, John Sinclair

**Affiliations:** ^1^​ Department of Medicine, University of Cambridge, Cambridge, UK; ^†^​ Present address: Public Health Agency of Sweden, Solna, Sweden

**Keywords:** cytomegalovirus, CTCF, chromatin, latency, viral gene expression, virus-host interactions

## Abstract

Viral latency is an active process during which the host cell environment is optimized for latent carriage and reactivation. This requires control of both viral and host gene promoters and enhancers often at the level of chromatin, and several viruses co-opt the chromatin organiser CTCF to control gene expression during latency. While CTCF has a role in the latencies of alpha- and gamma-herpesviruses, it was not known whether CTCF played a role in the latency of the beta-herpesvirus human cytomegalovirus (HCMV). Here, we show that HCMV latency is associated with increased CTCF expression and CTCF binding to the viral major lytic promoter, the major immediate early promoter (MIEP). This increase in CTCF binding is dependent on the virally encoded G protein coupled receptor, US28, and contributes to suppression of MIEP-driven transcription, a hallmark of latency. Furthermore, we show that latency-associated upregulation of CTCF represses expression of the neutrophil chemoattractants S100A8 and S100A9 which we have previously shown are downregulated during HCMV latency. As with downregulation of the MIEP, CTCF binding to the enhancer region of S100A8/A9 drives their suppression, again in a US28-dependent manner. Taken together, we identify CTCF upregulation as an important mechanism for optimizing latent carriage of HCMV at both the levels of viral and cellular gene expression.

## Introduction

Human cytomegalovirus (HCMV) latency requires the epigenetic repression of transcription from the major immediate early promoter/enhancer region (MIEP) in order to prevent extensive lytic gene expression [1]. One important site of HCMV latency is in cells of the early myeloid lineage [2–5], where a variety of host and viral factors mediate the repressive chromatin structure around the MIEP [1, 6–12]. However, while immediate early (IE) gene expression is suppressed during latency, other viral genes are expressed [7, 13–18], suggesting that latency is distinct from viral quiescence. Indeed, several latency-associated gene products have been ascribed functions during HCMV latency, including immune evasion [19–21], PML body dispersion [22], and modulation of key cellular signalling pathways in order to suppress IE gene expression [12, 23–27].

The CCCTC-binding protein CTCF is a well-recognised chromatin and genome organiser that can positively or negatively regulate transcription, as well as insulate enhancer regions from promoter regions, associated with a diverse range of cellular functions [28]. CTCF can also bind the genomes of DNA viruses and integrated retroviruses, resulting in changes in viral gene expression [29–32]. CTCF has been shown to be important in the regulation of latency and reactivation of the herpesviruses HSV-1, EBV, and KSHV [33–41], and, interestingly, CTCF can also bind within the IE region of the HCMV genome [42]. During lytic infection, CTCF binds to the first intron of the IE1/IE2 locus to repress IE gene expression [42] and, thus, we hypothesised that CTCF binding to this region may be important during latency, when IE transcription must be repressed.

Here, we show that latent infection of monocytes by HCMV increases CTCF expression and that the latency-associated viral gene US28 is sufficient for this upregulation. Similarly, CTCF occupancy on the MIEP is enriched during latency in a US28-dependent manner, helping to mediate repression of the MIEP in myeloid cells. Since CTCF regulates host gene expression [43], we also examined the effect of CTCF upregulation in latent monocytes on expression of the chemoattractants S100A8 and S100A9 which we have previously shown are downregulated during HCMV latency to help evade neutrophil killing [44]. We show that latent infection drives increased CTCF occupancy on the enhancer region of these genes, and that US28 expressed in isolation in myeloid cells is able to drive decreased S100A8 and S100A9 secretion. Overall, we identify that CTCF is vital to HCMV latency-associated processes, including control of both host and viral gene expression.

## Methods

### Cells

All cells were maintained at 37 °C in a 5 % CO_2_ atmosphere. THP-1 cells (ECACC 88081201) were cultured in RPMI-1640 media (Sigma) supplemented with 10 % heat-inactivated foetal bovine serum (FBS; PAN Biotech), 100 U ml^−1^ penicillin and 100 µg ml^−1^ streptomycin (Sigma), and 0.05 mM 2-mercaptoethanol (Gibco). The 293T cells (ECACC 12022001) were maintained in DMEM (Sigma) supplemented with 10 % heat-inactivated FBS but without penicillin or streptomycin. Primary CD14^+^ monocytes were isolated from apheresis cones (NHS Blood and Transplant Service) or from peripheral blood taken from healthy volunteers as previously described [45]. Briefly, CD14^+^ monocytes were isolated from total peripheral blood mononuclear cells (PBMC) by magnetic-activated cell sorting (MACS) using CD14^+^ microbeads (Miltenyi Biotech). The monocytes were plated on tissue culture dishes (Corning) in X-Vivo 15 media (Lonza) supplemented with 2 mM l-glutamine.

### Plasmids

pHRsinGKpuro and pHRsinUbEm lentiviral expression vectors were a kind gift from Dr D van den Boomen, University of Cambridge. The sequence encoding US28 was cloned into pHRsinUbEm using the BamHI and NotI sites. The lentiviral expression vector encoding CTCFshRNA and puromycin resistance was purchased from Santa Cruz Biotechnology. The sequence encoding CTCF was cloned into pHRsinGKpuro using the EcoRI and XbaI/SpeI restriction sites.

### Lentivirus production and transduction

Generation of lentiviral particles was conducted generally in line with the Broad Institute Protocols. The 293T cells were seeded into 6-well plates at 5×10^5^ cells per well. Approximately 6 h later, 1250 ng of lentiviral expression vector, 625 ng of lentiviral packaging vector psPAX and 625 ng of lentiviral envelope vector pMD.2G (both gifts from S. Karniely, Kimron Veterinary Institute, Israel) were transfected into 293T cells using transfection reagent FuGene6 (Promega) according to the manufacturer’s instructions. The next morning, the media was aspirated and replaced with 2.5 ml RPMI supplemented with 30 % FBS. On the following morning, the supernatants containing lentiviral particles, plus a non-transfected control supernatant, were aspirated and centrifuged for 10 min at 1500 ***g*** to remove as many 293T cells as possible, and the media refreshed on the remaining adherent 293T cells. Supernatants were kept on ice while 2.5×10^5^ THP-1 cells were pelleted, and then resuspended in the lentiviral or control supernatant in a 6-well plate (one well per supernatant). Polybrene was added to the cells at 2 µg ml^−1^ and the cells were then centrifuged in the plate at 600 ***g*** for 45 min, and incubated overnight at 37 °C/5 % CO_2_. The following morning, this process was repeated in order to give cells two ‘doses’ of lentiviral supernatants. On the fifth morning, the transduced THP-1 cells were pelleted and resuspended in fresh RPMI supplemented with 10 % FBS. For vectors encoding puromycin resistance (shRNA CTCF and empty vector control), selection with puromycin (2 µg ml^−1^, Sigma) began 2 days after removal of lentiviral supernatants, and the selective media was refreshed every 2 days until all THP-1 cells which had been incubated with the non-transfected control supernatant were dead. For vectors encoding the green fluorescent protein Emerald (pHRsinUbEm US28 and empty vector control), Emerald positive cells were sorted using a BD FACSAriaIII instrument.

### Human cytomegaloviruses

Infection of monocytes was carried out at a multiplicity of infection (MOI) of three as determined by titration on RPE-1 cells. TB40/E*gfp* [46] was a gift from E.A. Murphy, SUNY Upstate Medical University. Titan WT and Titan ΔUS28 have been described previously [12].

Ultra-violet light (UV) inactivation of virus was performed by placing a 100 µl aliquot of virus in one well of a 24-well plate and placing this within 10 cm of a UV germicidal (254 nm) lamp for 15 min, which routinely results in no detectable IE gene expression upon infection of fibroblasts.

### Cell sorting

GFP positive latently infected monocytes were separated from GFP negative bystander monocytes at three dpi using a BD FACSAria III instrument.

### Western blotting

Cells were lysed directly in Laemmli Buffer and separated by SDS-PAGE. Following transfer to nitrocellulose, the membrane was blocked in 5 % milk in tris buffered saline (TBS) with 0.1 % Tween-20. Antibodies used: anti-CTCF (Abcam ab70303), anti-GAPDH (Abcam ab9485), anti-beta actin (Abcam ab6276).

### Chromatin immunoprecipitation (ChIP)

Chromatin immunoprecipitation was performed using the Imprint ChIP kit (Sigma) according to the manufacturer’s instructions using a ChIP grade isotype control or CTCF specific antibody (Abcam ab70303). Primers for analysing enrichment are shown in Table 1. Quantitative PCR was performed using New England Biotech LUNA SYBR Green qPCR reagents (Intron 1 target) or Qiagen Quantitect Probe RT-qPCR reagents (Transcription start site (TSS) target) with the probe (FAM)TGGGAGTTTGTTTTGGCACCAAA(TAM).

**Table 1.** List of primers used in this study. Application (App^n^) is denoted by ‘Qu’ for RT-qPCR, ‘Ch’ for ChIP. All primer sequences are 5′−3′

Target	App^n^	Forward	Reverse
IE1	Qu	GTCCTGACAGAACTCGTCAAA	TAAAGGCGCCAGTGAATTTTTCTTC
GAPDH	Qu	TGCACCACCAACTGCTTAGC	GGCATGGACTGTGGTCATGAG
US28	Qu	AATCGTTGCGGTGTCTCAGT	TGGTACGGCAGCCAAAAGAT
CTCF	Qu	ATGTGCGATTACGCCAGTGTA	TGAAACGGACGCTCTCCAGTA
GAPDH promoter	Qu	CGGCTACTAGCGGTTTTACG	AAGAAGATGCGGCTGACTGT
UL44 promoter	Qu	AACCTGAGCGTGTTTGTG	CGTGCAAGTCTCGACTAAG
S100A8/9 enhancer	Ch	GGACATGGGGCAACCTAGAG	GGCTCCACAGGCATTGAGTA
MIEP (intron 1 target)	Ch	GGAGCTTCCACATCCGAGCC	CAGACACATACCCTACCGCC
MIEP (TSS target)	Ch	CCAAGTCTCCACCCCATTGAC	GACATTTTGGAAAGTCCCGTTG

### DNA and RNA extraction, reverse transcription, and quantitative PCR

RNA was extracted and purified using Direct-Zol RNA MiniPrep kit (Zymo Research) according to the manufacturer’s instructions. QuantiTect SYBR Green RT-PCR Kit reagents (Qiagen) were used for RT-qPCR. Glyceraldehyde phosphate dehydrogenase (GAPDH) was used as a reference gene and relative gene expression was analysed using ΔC_t_ or ΔΔC_t_ values. Primer sequences are given in Table 1.

For analysis of viral genomes, cells were pelleted and washed with citrate buffer (40 mM sodium citrate, 10 mM KCl, 135 mM NaCl, pH 3.0) to remove externally bound virions. Subsequently, cells were washed with PBS, then resuspended in solution A (100 mM KCl, 10 mM Tris-HCl pH8.3 2.5 mM MgCl) was added followed by an equal volume of solution B (10 mM Tris-HCl pH8.3, 2.5 mM MgCl 1 % Tween 20, 1 % NP-40, 0.4 mg ml^−1^ proteinase K). The lysed cells were heated at 60 °C for 1 h then 95 °C for ten minutes. The DNA preparation was then analysed by qPCR using LUNA reagents (New England Biotech) using the UL44 non-transcribed promoter region as the viral target, and the GAPDH non-transcribed promotor region to correct for total DNA levels. Primer sequences are provided in Table 1.

### Transfection and nucleofection

For analysis of the effect of CTCF on IE gene expression and levels of viral genome, THP-1 cells were transfected, by nucleofection, with the CTCF lentiviral overexpression vector or empty vector, using the Lonza Nucleofector Kit R. These cells were infected with HCMV 24 h later. Alternatively, THP-1 cells transduced with CTCF shRNA vector or control vector (described above in Lentivirus production and transduction) were infected with HCMV.

For analysis of MIEP activity, THP-1 cells were transfected, by nucleofection, with an MIEP-luciferase construct [47], as well as an SV40-Luciferase (Renilla) construct (pRL, Promega) as a transfection control, as well as with a CTCF vector or empty vector as described above. After 48 h, luciferase assays were performed using DualLuciferase Reporter Assay System (Promega), following manufacturer’s protocol, using a GloMax−96 Microplate Luminometer.

For analysis of the activity of the S100A8/A9 enhancer, THP-1 cells were transfected with plasmid pLightSwitchLR (SwitchGearGenomics, Active Motif) in which the S100A8, A9 and A12 CTCF responsive enhancer (GRCh37:chr1 : 153366418–153366818) had been cloned as well as a control plasmid driving the expression of beta-galactosidase, using the transfection reagent FuGENE HD (Active Motif). Luciferase and beta-galactosidase accumulation were then measured as previously published [48].

### S100A8/A9 ELISA

Detection of S100A8/A9 heterodimers in supernatant was via enzyme-linked immunosorbent assay (ELISA) (BioLegend) following instructions from the manufacturer.

### CTCF binding site prediction

The sequence spanning the entire HCMV major immediate early region (NC_006273.2 nucleotides 170,568–176,978) was inputted into CTCFBSDB2.0 [49] to identify potential CTCF binding sites.

## Results

### CTCF binds the major immediate early region during HCMV latency

CTCF is an important regulator of alpha- and gamma-herpesvirus latency [29, 33, 35, 37, 38], but the role of CTCF in the latency of the beta-herpesvirus HCMV has not been described. Previous results have shown that CTCF can bind and suppress MIE gene expression during lytic infection of fibroblasts [42], so we predicted that this function could be of major importance in latently infected monocytes where general suppression of major IE expression occurs [50–53].

**Fig. 1.**
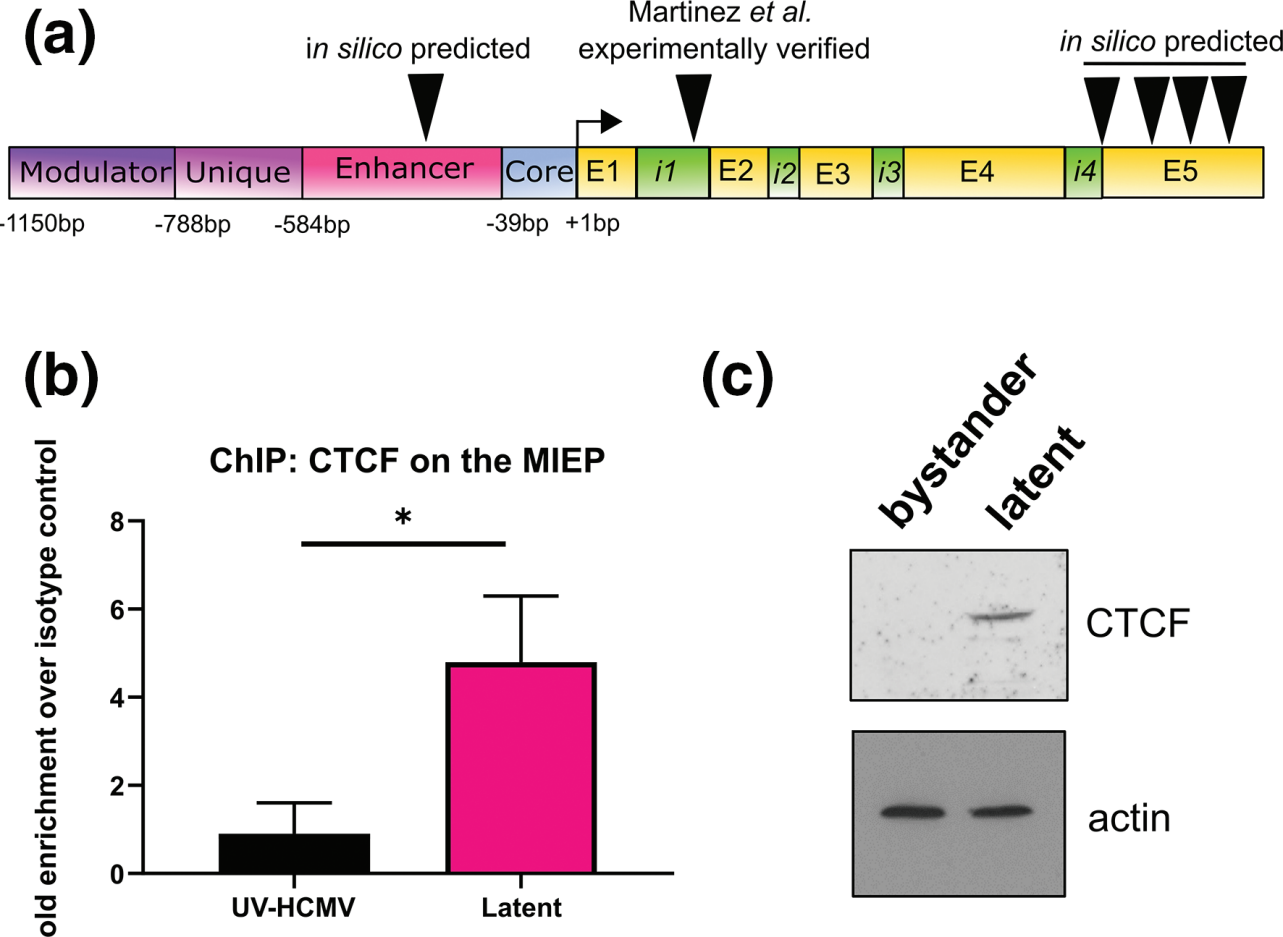
CTCF binds the MIEP during HCMV latency. (**a**) Schematic of the MIE locus, indicating the positions of the predicted and experimentally verified CTCF binding sites. Exons and introns are denoted by the letters E and i, respectively. (**b**) Primary CD14+ monocytes were treated with UV-inactivated virus, or infected with HCMV (strain Titan, MOI 3). At six dpi, cells were fixed with formaldehyde and subjected to chromatin extraction and analysis by ChIP using a CTCF antibody or isotype control. Fold enrichment of MIEP sequences is shown with respect to isotype control. Statistical analysis by two-tailed *t*-test, * indicates *P* <0.05. (**c**) Primary CD14+ monocytes were infected with HCMV strain TB40/Egfp at MOI 3. At three dpi, GFP positive cells (latent) were separated from GFP negative cells (bystander) by FACS. CTCF protein level was then analysed by Western blot, using actin as a loading control.

We began by identifying potential CTCF binding sites on the entire MIEP region using CTCFBSDB2.0 [49], which identified six potential binding sites (Fig. 1a, full sequences presented in Fig. S1, available in the online version of this article). One of these, within intron 1, has previously been experimentally verified during lytic infection of fibroblasts [42]. We next determined whether CTCF binds the MIEP region during latency by chromatin immunoprecipitation (ChIP) in latently infected CD14^+^ monocytes, one important cellular site of HCMV latency [2, 54]. Using primers targeted to the major IE transcription start site, we found that latently infected monocytes had increased CTCF occupancy on the MIEP region as predicted (Fig. 1b), indicating that CTCF might play a role in the control of MIEP activity during latency. Since these primers are in range of both the previously validated CTCF site (~920 bp separation), and the predicted enhancer site (~170 bp separation), we could not draw conclusions about which of these sites was functional during latency in this analysis. We then analysed CTCF protein levels in latently infected monocytes, since an increase in latency-associated occupancy of CTCF on the MIEP could be a result of an increase in CTCF levels during latency. To analyse this, we isolated latent populations of monocytes experimentally infected with the HCMV strain TB40/E*gfp* using FACS, separating latently infected cells from bystander cells [44]. We found that latently infected monocytes have greatly increased levels of CTCF protein compared with bystander uninfected monocytes (Fig. 1c). This suggests that CTCF levels are increased by HCMV during latency which likely promotes CTCF binding to the MIEP, and potentially other promoters as well.

### CTCF represses the major immediate early promoter during HCMV latency

**Fig. 2.**
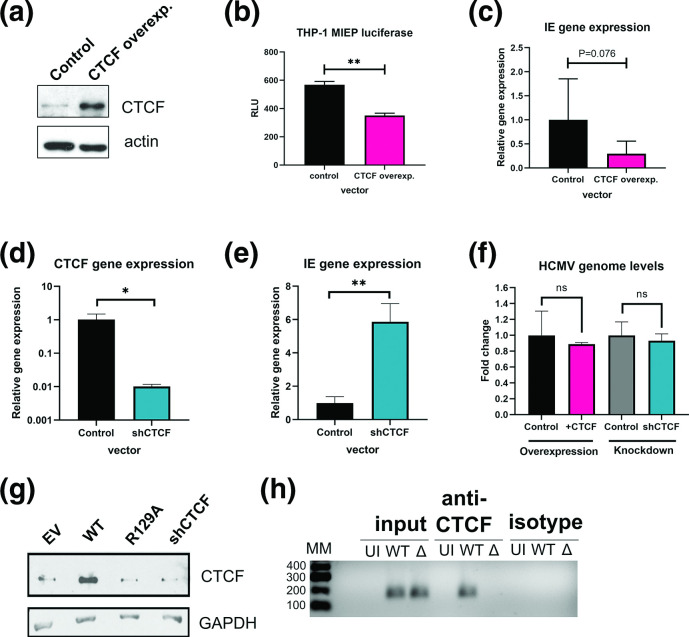
CTCF binds and represses the MIEP in a US28 dependent manner. (**a**) THP-1 cells were transfected with control or CTCF overexpression vector and after 2 days cell lysates were analysed for CTCF overexpression by Western blot using actin as a loading control (b) THP-1 cells were transfected with the control or CTCF overexpression vectors as (**a**) along with an MIEP luciferase vector and transfection control renilla vector. After 2 days, luciferase activity was measured and is quantified as relative light units (RLU). (**c**) THP-1 cells transfected with CTCF overexpression or control vector were then infected with HCMV Titan WT. At four dpi, total RNA was harvested and analysed for IE gene expression. (**d**) THP-1 cells were transduced with control lentivirus or CTCF shRNA lentivirus. CTCF knockdown was confirmed by RT-qPCR for CTCF mRNA. (**e**) Cells from (**d**) were infected as per (**c**) and analysed for IE gene expression. (**f**) THP-1 cells transfected with control or CTCF overexpression vector as (**a**) or transduced with control or CTCF shRNA lentivirus as (**d**) were infected with HCMV Titan WT. After 16 h, cells were pelleted, washed with citrate buffer to remove externally bound virions, and analysed for HCMV genome levels by qPCR. Fold change in HCMV genome levels is presented with respect to the relevant control vectors. (**g**) THP-1 cells transduced with Empty vector (EV), US28-WT, US28-R129A, and CTCF-targeting-shRNA were lysed and CTCF protein was analysed by Western blot, using GAPDH as a loading control. (**h**) Primary CD14+ monocytes were left uninfected, infected with Titan WT (WT), or Titan ΔUS28 (Δ). At six dpi, cells were fixed with formaldehyde and subject to chromatin extraction and analysis by ChIP using a CTCF antibody or isotype control. Following qPCR for the MIEP region, PCR products from input, anti-CTCF antibody, and isotype control precipitations were separated on a 1.5 % agarose gel. Molecular mass markers (MM) are indicated in numbers of base pairs. The results of statistical analysis by two-tailed *t*-test are indicated, **, *P* <0.01; *, *P* <0.05, or *P* values are given numerically.

**Fig. 3.**
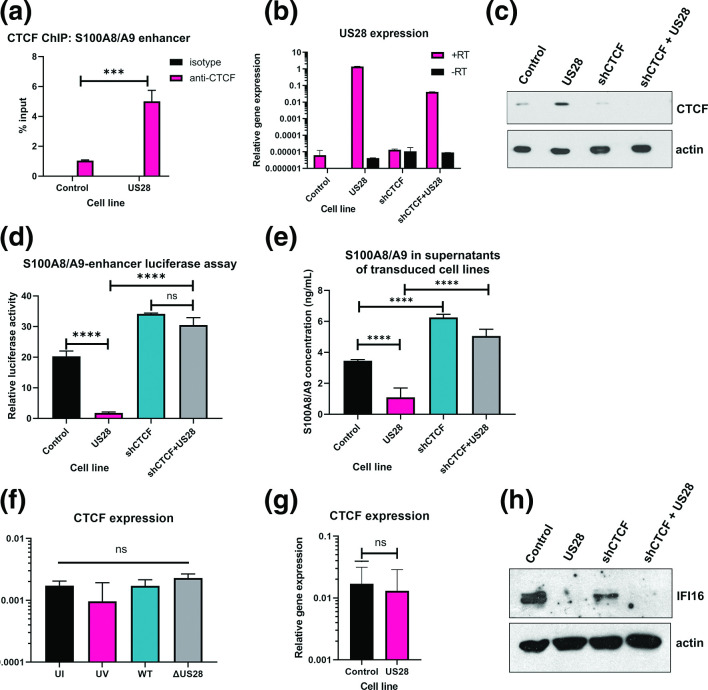
US28 mediates S100A8/A9 downregulation via CTCF. (**a**) Enrichment of CTCF on the S100A8/A9 enhancer in control and US28-expressing THP-1 cells was detected by ChIP analysis using a CTCF or isotype control antibody and is expressed as % input. Statistical analysis by two-tailed *t*-test, *** indicates *P* <0.001. (**b**) RT-qPCR analysis of US28 expression in transduced cell lines, using GAPDH as housekeeping control. Results are shown for reactions with reverse transcriptase (+RT) and without reverse transcriptase (-RT) to control for gDNA contamination. (**c**) Western blot analysis of CTCF expression in control or US28-expressing THP-1 cell lines, using actin as a loading control. (**d**) S100A8/A9 enhancer luciferase assay in cells from B. (**e**) S100A8/A9 ELISA in supernatants from cells in B. (**f**) CTCF expression was detected by RT-qPCR in monocytes left uninfected (UI) or treated with UV-inactivated HCMV (UV), or infected with HCMV Titan WT (WT), or Titan ΔUS28 (ΔUS28) for 6 days. (**g**) CTCF expression was detected by RT-qPCR in control and US28-expressing THP-1 cells (h) IFI16 protein expression in cells from B. Statistical analysis for C, D by one-way ANOVA followed by Tukey’s multiple comparison test, *** indicates *P* <0.001, *****P <0.0001, ns=not significant.

To elucidate the functional outcome of CTCF upregulation during latency, we analysed whether MIEP activity is affected by CTCF expression in myeloid cells. To do this we interrogated the effect of CTCF on MIEP activity and IE gene expression in THP-1 cells, a myelomonocytic cell line used by many as a model of HCMV latency [21, 55]. We began by overexpressing CTCF by plasmid transfection (Fig. 2a) and analysing the effect of CTCF overexpression on MIEP activity using an MIEP-luciferase reporter construct [47]. As predicted, overexpression of CTCF decreased MIEP activity (Fig. 2b). To confirm this result, we also latently infected THP-1 cells transiently overexpressing CTCF and measured IE gene expression. We found a trend towards decreased viral IE gene expression in infected cells overexpressing CTCF (Fig. 2c). We then took the opposite approach and knocked down CTCF by transduction of THP-1 cells with lentivirus encoding CTCF shRNA. After validating the successful knockdown of CTCF shRNA by RT-qPCR (Fig. 2d) and Western blot (Figs. 2g and 3c), we infected control and CTCF knockdown cells with HCMV-WT. As predicted, we saw significantly increased IE gene expression in the CTCF knockdown cells. To exclude the possibility that interfering with CTCF expression affects HCMV binding/entry and the earliest events of infection, rather than IE gene expression itself, we analysed HCMV genome levels in CTCF overxpressing, CTCF knockdown, and relevant control cells. This showed that depletion or overexpression of CTCF had no effect on uptake of levels of HCMV DNA (Fig. 2f). These results are consistent with the known effects of CTCF during lytic infection of fibroblasts [42] and confirms that CTCF levels are also important for regulation of IE gene expression during the establishment and/or maintenance of latency.

### US28 mediates upregulation of CTCF and increased binding to the MIEP during latent infection

We then speculated that the virally encoded G protein coupled receptor US28 may play a role in CTCF upregulation, because (i) CTCF was previously shown to repress MIEP activity during lytic infection and (ii) a key role of US28 during latency is the negative regulation of the MIEP [11, 12, 42]. Having established that CTCF protein levels are increased in latently infected monocytes, we then investigated whether US28, in isolation, is sufficient for CTCF upregulation. To do this, we analysed lysates from THP-1 cells expressing US28-WT in isolation (Fig. 2d) [12]. As controls, we used THP-1 cells expressing US28-R129A (a signalling mutant deficient for many latency associated functions [12, 27, 56]), the relevant empty vector, and a CTCF-targeting short hairpin RNA (shCTCF). We found that only US28-WT-expressing THP-1 cells had increased CTCF protein, indicating that US28 is sufficient for CTCF upregulation in myeloid cells, and that this effect is dependent on US28 signalling.

Since US28 is sufficient for CTCF upregulation in myeloid cells, we were interested in whether US28 is necessary for CTCF binding to the MIEP during latency. To do this, we infected CD14^+^ monocytes with HCMV-WT, or HCMV-ΔUS28. Using a second ChIP-semi-quantitative PCR analysis, this time using primers targeted to the previously published CTCF binding site, we again found enrichment of CTCF on the MIEP in latently infected CD14^+^ monocytes as before, but no enrichment of CTCF on the MIEP in monocytes infected with HCMV-ΔUS28 (Fig. 2h). These results suggest that US28 increases binding of CTCF to the MIEP during HCMV latency to reduce IE gene expression. Taken together with our results using cells expressing US28 in isolation (Fig. 2g), these data suggest that CTCF binding at the MIEP during latency could be driven by US28-mediated upregulation of CTCF.

### CTCF upregulation downregulates expression of S100A8 and A9 chemoattractants during latency

CTCF has been well documented effects on host transcription in addition to roles in viral gene regulation [28, 57–60]. We reasoned, therefore, that there may be additional effects of latency-associated CTCF upregulation on host gene expression, which may in turn be important for HCMV latency.

**Fig. 4.**
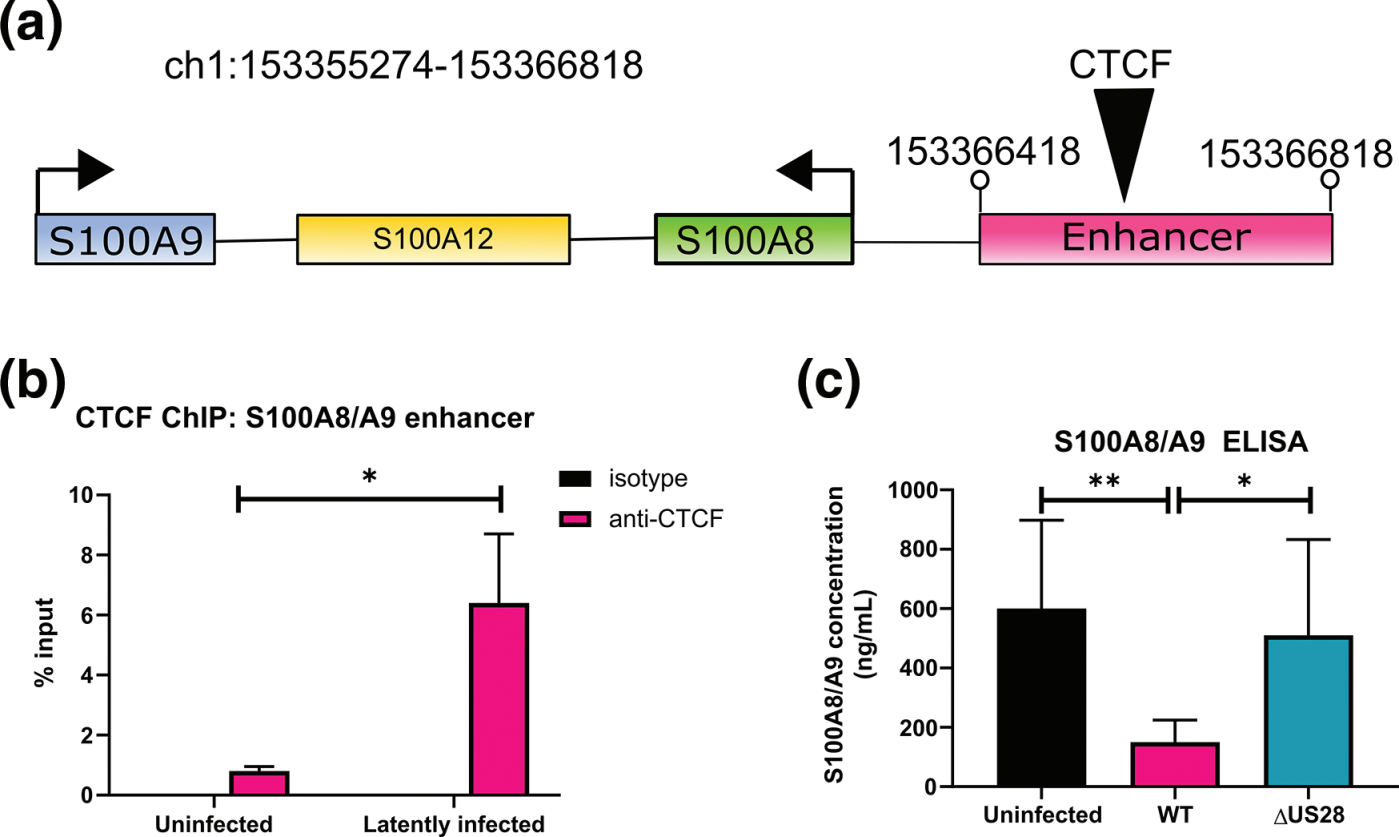
S100A8/A9 downregulation is associated with CTCF binding to an enhancer region. (**a**) Schematic showing the organisation of the S100A8, S100A9, and S100A12 gene region and enhancer region, which contains a CTCF binding site (black triangle). Genomic coordinates of the enhancer are given. (**b**) Enrichment of CTCF on the S100A8/A9 enhancer in sorted latently infected monocytes was detected by ChIP analysis of latently infected, or uninfected monocytes at six dpi using a CTCF or isotype control antibody. Statistical analysis by two-tailed *t*-test, * indicates *P* <0.05. (**c**) S100A8/A9 heterodimer concentration was measured in the supernatants from uninfected monocytes, monocytes infected with Titan WT or with Titan ΔUS28 at six dpi. Statistical analysis by one-way ANOVA followed by Tukey’s multiple comparison test, * indicates *P* <0.05, **P <0.01.

We have recently shown that the neutrophil chemoattractants S100A8 and S100A9 are downregulated during HCMV latency, resulting in the evasion of neutrophil killing [44], but the mechanism for this downregulation was unclear. We reasoned that the observed latency-associated changes in CTCF might also be involved in the downregulation of S100A8/A9 during latency because there is a predicted CTCF site in the enhancer region of the S100A8, S100A9, and S100A12 genomic locus [61, 62] (Fig. 4a). To investigate whether CTCF mediates S100A8/A9 downregulation, we began by analysing CTCF enrichment on the S100A8/A9 enhancer region by ChIP. We again purified latently infected CD14^+^ monocytes by FACS associated cell sorting and, as predicted, found that latently infected monocytes had increased CTCF enrichment on the S100A8/A9 enhancer region compared with uninfected monocytes (Fig. 4b), suggesting that CTCF could play a role in control of S100A8/A9 during latency.

Since US28 was sufficient for CTCF upregulation in myeloid cells (Fig. 2d), and was required for CTCF binding to the MIEP during latency (Fig. 2e), we hypothesised that S100A8/A9 downregulation would depend on US28. Consequently, we analysed S100A8/A9 heterodimer concentration in the supernatants taken from uninfected, HCMV-WT latently infected, and HCMV-ΔUS28 infected primary CD14^+^ monocytes. We found, as previously published [44], that supernatants from latently infected monocytes contained lower amounts of S100A8/A9 heterodimers than uninfected monocytes (Fig. 4c). However, deletion of US28 abrogated this effect, consistent with the view that US28, via CTCF upregulation, can modulate S100A8 and S100A9 expression.

### US28 is sufficient for CTCF-mediated downregulation of S100A8/A9

To support our observations of US28 directed control of S100A8/A9 expression, via CTCF, during latency, we used US28-expressing THP-1 cells [27] to interrogate the molecular mechanisms at play. We first analysed CTCF enrichment on the S100A8/A9 enhancer in the presence or absence of US28. Consistent with our observations during experimental latency (Fig. 4b), US28, when expressed in isolation, resulted in greater occupancy of CTCF on the S100A8/A9 enhancer (Fig. 3a). To confirm that this effect was dependent on CTCF, we generated THP-1 cells expressing US28 alone, a short hairpin RNA (shRNA) targeting CTCF (shCTCF), as well as cells expressing both US28 and shCTCF together, or their control vectors only. We confirmed US28 expression by RT-qPCR (Fig. 3b). We then confirmed that, in these cells, CTCF protein is increased by US28, as seen before (Fig. 2d), and that the shCTCF resulted in knockdown of CTCF expression (Fig. 3c). Subsequently, we transfected an S100A8/A9 enhancer luciferase reporter vector into these cells to analyse the effect of US28 on the enhancer activity, and any dependence of this effect on CTCF. In cells where US28 was expressed in isolation, the activity of the enhancer was suppressed (Fig. 3d). However, knocking down endogenous CTCF in US28 expressing cells abrogated this effect, indicating that US28 requires CTCF to drive enhancer repression. These effects were recapitulated using ELISA based assays of S100A8/A9 heterodimers in the supernatants of these cells, in that US28-expressing THP-1 cells had lower levels of S100A8/A9 heterodimers compared to control cells. Similarly, this downregulation of secreted S100A8/A9 was abrogated when CTCF was knocked down (Fig. 3e). Our results suggest a potential mechanism by which HCMV latent infection downregulates S100A8/A9: repressive CTCF binding to the enhancer region of the S100A8/A9 genomic locus is increased during HCMV latency, likely mediated by the viral gene product US28. Exactly how US28 modulates CTCF expression during latent infection of monocytes is not completely clear, though it appears to be post-transcriptional as latent infection of monocytes or overexpression of US28 in THP-1 cells has little effect on levels of CTCF RNA (Fig. 3f, g, respectively), and awaits further investigation.

Finally, as US28 is known to manipulate expression of many different host genes, by a variety of mechanisms [12, 25, 27, 56], we wanted to know whether US28-mediated upregulation of CTCF could be a general mechanism by which US28 controls host gene expression or only used to modulate specific host and viral gene expression. We used IFI16, a host gene we have shown to be repressed by US28 during latency [27], to test this hypothesis. As previously shown, US28 expression in isolation downregulated IFI16 (Fig. 3h) . However, this could not be rescued by the knockdown of CTCF via shRNA. This indicates that CTCF upregulation by US28 does not significantly contribute to US28-mediated downregulation of IFI16. Overall, these data argue that US28-mediated changes in cellular gene expression during latency are likely mediated by a number of different mechanisms besides US28 upregulation of CTCF.

## Discussion

Viral latency is increasingly accepted to involve virus-directed modulation of host cells for the benefit of latent carriage and reactivation. During latency, HCMV suppresses its lytic transcription programme whilst optimising the cellular environment and evading host immunity [23, 44, 50, 63, 64]. Here, we have identified upregulation of CTCF as an important feature of HCMV latency that likely controls both host and viral gene expression.

We found that CTCF protein expression was higher in latent monocytes compared with uninfected bystanders, a phenomenon that can likely be attributed to the HCMV encoded G protein coupled receptor, US28. This viral gene product is expressed during latency and is essential for the establishment and maintenance of latency in multiple experimental models [11, 12, 25, 56]. US28 suppresses the MIEP via host genes including MAPK, STAT3, c-fos, NF-κB, and IFI16 [12, 25, 27, 56]; here we now show that US28 also directs MIEP suppression by enhancing CTCF binding to the MIEP.

Our ChIP analyses clearly showed increased recruitment of CTCF to the MIEP region in latently infected monocytes which was only observed if US28 was present in the infecting virus. Our *in silico* predictions for potential CTCF binding sites in the MIEP region identified the previously published CTCF binding site in intron 1 of the major IE transcription unit [42] but also identified a putative CTCF binding site in the MIEP enhancer (see Fig. 1a). Interestingly, in our transfection assays which used MIEP reporters carrying the enhancer but lacking intron 1 of the MIEP region, we still observed the ability of CTCF to negatively regulate the MIEP, consistent with the view that the putative CTCF binding site within the enhancer may also be functioning as a site of CTCF-mediated repression of the MIEP. It would be interesting to establish whether US28-mediated regulation of CTCF impacts similarly latency establishment, long-term latency maintenance, and reactivation from latency. However, because of the multitude of effects of US28 on the latency process, this is not trivial to investigate.

This US28-mediated upregulation of CTCF also played a role in controlling cellular gene expression in that it mediated the downregulation of the neutrophil chemoattractants S100A8 and S100A9. We found that CTCF binds to the distal enhancer of this gene cluster during latency and that modulation of CTCF expression in isolation controls S100A8/A9 expression, and confirmed that this was, in turn, dependent on US28. Our previous work has shown that US28 downregulates cellular interferon-stimulated genes [27], and, here, we have found that US28 is also involved in the downregulation of secreted chemokines. Clearly, US28 has multiple roles during HCMV latency, as it does during lytic infection [65].

CTCF has well-established roles in the latencies of the herpesviruses HSV-1, EBV, and KSHV [33–41], where particular attention has been paid to control of viral transcription. Many of these studies have used high throughput technologies such as ChIP-Seq and Hi-C to elucidate complex interactions and identify distal chromatin contacts that are important for the virus. Our view is that the role of CTCF during latent and lytic HCMV infection should now also be expanded using comprehensive analyses of chromatin architecture, and CTCF/cohesin interactions during HCMV lytic and latent infections, including reactivation. This may provide information on genome maintenance during latency, and higher order transcriptional control of HCMV infection, topics which are currently not well understood in the HCMV field.

## Supplementary Data

Supplementary material 1
