# Supplementary material for: Regulation of host and viral promoters during human cytomegalovirus latency via US28 and CTCF
Source: J Gen Virol. 2021 May 27;102(5):001609. doi: 10.1099/jgv.0.001609 (PMC8295918; doi:10.1099/jgv.0.001609)
Supplement: Supplementary material 1 [file jgv-102-1609-s001.pdf]

Supplementary Figure 1. The major immediate early locus (NC_006273.2 nucleotides 170,568-176,978) is displayed with annotated exons, the transcription start site (TSS), and the CTCFBSDB2.0-predicted CTCF binding sites, using the nomenclature of the database to describe each site.

5′ GAGACCAGAGACGGAGATGTATAAAAAGCTACTTTTATTAAACAGCATTCTCACCACACGTTAATACTGTCACGGGAAATCACTATGTACAAGA 95
3′ CTCTGGTCTCTGCCTCTACATATTTTTCGATGAAAATAATTTGTCGTAAGAGTGGTGTGCAATTATGACAGTGCCCTTTAGTGATACATGTTCT

GTCCATGTCTCTTTCCAGTTTCTCACTTACTGAGATTTGTTCCTCAGGTCCTGGATGGCTGCCTCGATGGCCAGGCTCAGGGTGTCCAGGTCTTC 190
CAGGTACAGAGAAAGGTCAAAGAGTGAATGACTCTAAACAAGGAGTCCAGGACCTACCGACGGAGCTACCGGTCCGAGTCCCACAGGTCCAGAAG
Exon 5

GGGAGGGGTCTCGGTGGGCTTCTCAAACTGCCCCACGGCGTAGGCCTTCGCGGCCGTCTCGTAGATAGGCAGCATGAACCCACCCTGGTTGGTGG 285
CCCTCCCCAGAGCCACCCGAAGAGTTTGACGGGGTGCCGCATCCGGAAGCGCCGGCAGAGCATCTATCCGTCGTACTTGGGTGGGACCAACCACC
Exon 5

AGAAGATGCGCACCATGACCTGTTTGGGAAACTTTTGCATCAGGGGCAGGCACAGGTTGAGAGCGCCCAACAGGTCCACGGGGGTGGCAGCGTGG 380
TCTTCTACGCGTGGTACTGGACAAACCCTTTGAAAACGTAGTCCCCGTCCGTGTCCAACTCTCGCGGGTTGTCCAGGTGCCCCCACCGTCGCACC
Exon 5

ATAATCATGTTGCGGTAATCAGAAGAGCGGGGGCACAATTGGTGGGTGTGCAGTTCTTTGAGGTTCCACACGGCCTTGACACCGTCGTTACAAGC 475
TATTAGTACAACGCCATTAGTCTTCTCGCCCCCGTGTTAACCACCCACACGTCAAGAAACTCCAAGGTGTGCCGGAACTGTGGCAGCAATGTTCG
Exon 5
EMBL_M2 CTCF motif

ATCGGCCGTGCGCTGCGCCACATCAGGAGGGTGTGTCACAGGCATAGTGTGCTCCATGAGGAAGGGAGTGGAGAGGGCCAGGTTGCACATGCTAC 570
TAGCCGGCACGCGACGCGGTGTAGTCCTCCCACACAGTGTCCGTATCACACGAGGTACTCCTTCCCTCACCTCTCCCGGTCCAACGTGTACGATG
Exon 5

CCAGGCGACACCGTACCTGATCCACCTCATTCTTCACTTCATGATTGCGGGTGTAGATGATCTGAATGCCCTTGTTGTTCACCTGCATGGTTTTG 665
GGTCCGCTGTGGCATGGACTAGGTGGAGTAAGAAGTGAAGTACTAACGCCCACATCTACTAGACTTACGGGAACAACAAGTGGACGTACCAAAAC
Exon 5

CAAACTTTGATGGCCTCTTCTAACACTTGGTGCATACTAGGGATCATGAATGGCAGATTCTTGTACTCAAGAGAACGATTGGTGTGACGGAACAT 760
GTTTGAAACTACCGGAGAAGATTGTGAACCACGTATGATCCCTAGTACTTACCGTCTAAGAACATGAGTTCTCTTGCTAACCACACTGCCTTGTA
Exon 5

GCGGCTCACCTCGTCAATCTTGACGCGACCCCGCCGAGTCTGCACGTTGGGTGTGCAGAAGGGGGTGTTCTTATCTTTCATGATATTGCGCACCT 855
CGCCGAGTGGAGCAGTTAGAACTGCGCTGGGGCGGCTCAGACGTGCAACCCACACGTCTTCCCCCACAAGAATAGAAAGTACTATAACGCGTGGA
Exon 5
MIT_ML7 CTCF site

TCTCGTTGTCCAACTCGGAGATGCGTTTGCTCTTCTTCTTGCGAGGTCCGGTGCTCGCCCCGCCGCTGCTCTGATGGCCGCAGCTCAGCAGAGAG 950
AGAGCAACAGGTTGAGCCTCTACGCAAACGAGAAGAAGAACGCTCCAGGCCACGAGCGGGGCGGCGACGAGACTACCGGCGTCGAGTCGTCTCTC
Exon 5

GAGGAGGCCGCGCCACCAAAACCGCCGCGCCCATGGTGGCTCGAGGTCACGGATGCTCCTCCGCCACTGCTGCATTTCATCTCCTCGGACTCACT 1045
CTCCTCCGGCGCGGTGGTTTTGGCGGCGCGGGTACCACCGAGCTCCAGTGCCTACGAGGAGGCGGTGACGACGTAAAGTAGAGGAGCCTGAGTGA
Exon 5

CTCCGAGTCCGAAGCCGAACTGCAGGAGGAGGAAGACGAAGAGGAACTATCTTCATCGGGCCGGCCCAAAGGATCGGGAAGAGGAGGGTGGTTCA 1140
GAGGCTCAGGCTTCGGCTTGACGTCCTCCTCCTTCTGCTTCTCCTTGATAGAAGTAGCCCGGCCGGGTTTCCTAGCCCTTCTCCTCCCACCAAGT
Exon 5
REN_20 CTCF motif

TCTGGGAGAGCGGGTGCGTGGGAGAGGTCACTCGCGGCGTGCCGCTGCCGGTGGAAGGGGAAGACGCGGTAGCACCGCGGGTCTCGACTTCTTCA 1235
AGACCCTCTCGCCCACGCACCCTCTCCAGTGAGCGCCGCACGGCGACGGCCACCTTCCCCTTCTGCGCCATCGTGGCGCCCAGAGCTGAAGAAGT
Exon 5

CCCTGTTCTTCCTCGCTATCAGAGATCACGATACAGCCGGCGGTATCGATAATCTTGTTGCGGTACTGGATGGTAAAGTCGGGCTCGGGCTTGAT 1330
GGGACAAGAAGGAGCGATAGTCTCTAGTGCTATGTCGGCCGCCATAGCTATTAGAACAACGCCATGACCTACCATTTCAGCCCGAGCCCGAACTA
Exon 5

GTCTTCCTGTTTGATGAGGGGCAGCATGATAGGCGCGGGAGGCACGGGCGGTTTAATAATCACCTTGAAAGGACGCGTGGTTTTGCGCGGTTTCT 1425
CAGAAGGACAAACTACTCCCCGTCGTACTATCCGCGCCCTCCGTGCCCGCCAAATTATTAGTGGAACTTTCCTGCGCACCAAAACGCGCCAAAGA
Exon 5

TACGCGGGCTGAGCTCGGGAGTAGCGGATGCCCCGGGGAGAGGAGTGTTAGTAACCGCGACGCTGGTGGGGGTCGGCTTGTTAAGAGGGGCGCTG 1520
ATGCGCCCGACTCGAGCCCTCATCGCCTACGGGGCCCCTCTCCTCACAATCATTGGCGCTGCGACCACCCCCAGCCGAACAATTCTCCCCGCGAC
Exon 5

CTAACGCTGCAAGAGTGGGTTGTCAGCGTGGGGCCGGTGCTACTGGAATCGATACCGGCATGATTGACAGCCTGGGCGAGGATGTCACCTGATGG 1615
GATTGCGACGTTCTCACCCAACAGTCGCACCCCGGCCACGATGACCTTAGCTATGGCCGTACTAACTGTCGGACCCGCTCCTACAGTGGACTACC
Exon 5
MIT_LM23 CTCF motif

TGATAAGAAGACACGGGAGACTTAGTACGGTTTCACAGGCGTGACACGTTTATTGAGTAGGATTACAGAGTATAACATAGAGTATAATATAGAGT 1710
ACTATTCTTCTGTGCCCTCTGAATCATGCCAAAGTGTCCGCACTGTGCAAATAACTCATCCTAATGTCTCATATTGTATCTCATATTATATCTCA
MIT_LM23 CTCF motif

ATACAATAGTGACGTGGGATCCATAACAGTAACTGATATATATACAATAGTTTACTGGTCAGCCTTGCTTCTAGTCACCATAGGGTGGGTGCTCT 1805
TATGTTATCACTGCACCCTAGGTATTGTCATTGACTATATATATGTTATCAAATGACCAGTCGGAACGAAGATCAGTGGTATCCCACCCACGAGA
Exon 4

TGCCTCCAGAGGCGGTGGGTTCCTCAGCACCATCCTCCTCTTCCTCTGAGGCAACTTCCTCTATCTCAGACACTGGCTCAGACTTGACAGACACA 1900
ACGGAGGTCTCCGCCACCCAAGGAGTCGTGGTAGGAGGAGAAGGAGACTCCGTTGAAGGAGATAGAGTCTGTGACCGAGTCTGAACTGTCTGTGT
Exon 4

GTGTCCTCCCGCTCCTCCTGAGCACCCTCCTCCTGTTCCTCATCACTCTGTTCACTTTCTTCCTGATCACTGTTCTCAGCCACAATTACTGAGGA 1995
CACAGGAGGGCGAGGAGGACTCGTGGGAGGAGGACAAGGAGTAGTGAGACAAGTGAAAGAAGGACTAGTGACAAGAGTCGGTGTTAATGACTCCT
Exon 4

CAGAGGGATAGTCGCGGGTACAGGGGACTCTGGAGGTGACACCAGAGAATCAGAGGAGCTGGCACCAGCGGTGGCCAAAGTGTAGGCTACAATAG 2090
GTCTCCCTATCAGCGCCCATGTCCCCTGAGACCTCCACTGTGGTCTCTTAGTCTCCTCGACCGTGGTCGCCACCGGTTTCACATCCGATGTTATC
Exon 4

CCTCTTCCTCATCTGACTCCTCGGCGATGGCCCGTAGGTCATCCACACTAGGAGAGCAGACTCTCAAAGGATCGGCCCCCAGAATGTACTGGGCA 2185
GGAGAAGGAGTAGACTGAGGAGCCGCTACCGGGCATCCAGTAGGTGTGATCCTCTCGTCTGAGAGTTTCCTAGCCGGGGGTCTTACATGACCCGT
Exon 4

AAGACCTTCATGCAGATCTCCTCAATGCGGCGCTTCATTACACTGATAACCTCAGGCTTGGTTATCAGAGGCCGCTTGGCCAGCATCACACTAGT 2280
TTCTGGAAGTACGTCTAGAGGAGTTACGCCGCGAAGTAATGTGACTATTGGAGTCCGAACCAATAGTCTCCGGCGAACCGGTCGTAGTGTGATCA
Exon 4

CTCCTCTAAGACATAGCAGCACAGCACCCGACAGAACTCACTTAAGAGAGAGATGCCCCCGTACATGGTCATCATACAAGCGTCACTAGTGACCT 2375
GAGGAGATTCTGTATCGTCGTGTCGTGGGCTGTCTTGAGTGAATTCTCTCTCTACGGGGGCATGTACCAGTAGTATGTTCGCAGTGATCACTGGA
Exon 4

TGTACTCATTACACATTGTTTCCACACATGTAGTGAGGATATCCATAAATATGTGATCAATGTGCGTGAGCACCTTGTCTCTCTCCTCATCCAAA 2470
ACATGAGTAATGTGTAACAAAGGTGTGTACATCACTCCTATAGGTATTTATACACTAGTTACACGCACTCGTGGAACAGAGAGAGGAGTAGGTTT
Exon 4

ATCTTAAAGATTTTCTGGGCATAAGACATAATCTCATCAGGAGAGCACTGAGGCAAGTTCTGCAATGCCGCCATGGCCTGACTGCAGCCATTGGT 2565
TAGAATTTCTAAAAGACCCGTATTCTGTATTAGAGTAGTCCTCTCGTGACTCCGTTCAAGACGTTACGGCGGTACCGGACTGACGTCGGTAACCA
Exon 4

GGTCTTAGGGAAGGCTGAGTTCTTGGTAAAGAACTCTATATTCCTGTAGCACATATACATCATCTTTCTCCTAAGTTCATCCTTTTTAGCACGGG 2660
CCAGAATCCCTTCCGACTCAAGAACCATTTCTTGAGATATAAGGACATCGTGTATATGTAGTAGAAAGAGGATTCAAGTAGGAAAAATCGTGCCC
Exon 4

CCTTAGCCTGCAGTGCACCCCCCAACTTGTTAGCGGCGCCCTTGCTCACATCATGCAGCTCCTTAATACAAGCCATCCACATCTCCCGCTTATCC 2755
GGAATCGGACGTCACGTGGGGGGTTGAACAATCGCCGCGGGAACGAGTGTAGTACGTCGAGGAATTATGTTCGGTAGGTGTAGAGGGCGAATAGG
Exon 4

TCAGGTACAATGTAGTTCTCATACATGCTCTGCATAGTTAGCCCAATACACTTCATGTCCTCGAAAGGCTCATGAACCTTATCTAAGATATCTAA 2850
AGTCCATGTTACATCAAGAGTATGTACGAGACGTATCAATCGGGTTATGTGAAGTACAGGAGCTTTCCGAGTACTTGGAATAGATTCTATAGATT
Exon 4

GGCATTCTGCAAACATCCTCCCATCATATTAAAGGCGCCAGTGAATTTTTCTTCCGTCTGGGTATATTTTTTCAGCATGTGCTCCTTGATTCTAT 2945
CCGTAAGACGTTTGTAGGAGGGTAGTATAATTTCCGCGGTCACTTAAAAAGAAGGCAGACCCATATAAAAAAGTCGTACACGAGGAACTAAGATA
Exon 4

GCCGCACCATGTCCACTCGAACCTTAATCTGTTTGACTGTAGAGGAGGATAACAACACATATAAGTATCCGTCCTCCTGACTCATTTATCGCTAT 3040
CGGCGTGGTACAGGTGAGCTTGGAATTAGACAAACTGACATCTCCTCCTATTGTTGTGTATATTCATAGGCAGGAGGACTGAGTAAATAGCGATA
Exon 4

CTCGATGCCCCGCTCACATGCAAGAGTTAATCTTCACTCTATCTGACATACACAAGTAAATCCACGTCCCATGCAGGTTAGTATATATCACATAC 3135
GAGCTACGGGGCGAGTGTACGTTCTCAATTAGAAGTGAGATAGACTGTATGTGTTCATTTAGGTGCAGGGTACGTCCAATCATATATAGTGTATG

ATGTCAACAGACTTACCGAGTTCTGTCAGGACATCTTTTTCGGGGTTCTCGTTGCAATCCTCGGTCACTTGTTCAAAGGTTTTGAGGGATTCTTC 3230
TACAGTTGTCTGAATGGCTCAAGACAGTCCTGTAGAAAAAGCCCCAAGAGCAACGTTAGGAGCCAGTGAACAAGTTTCCAAAACTCCCTAAGAAG
Exon 3

GGCCAATTCTGGGAACAGCGGGTCTCCCAGGCTCAGCTGACTGTTAACCTCCTTCCTTAACATAGTCTGCAGGAACGTCGTGGCCTTGGTCACGG 3325
CCGGTTAAGACCCTTGTCGCCCAGAGGGTCCGAGTCGACTGACAATTGGAGGAAGGAATTGTATCAGACGTCCTTGCAGCACCGGAACCAGTGCC
Exon 3

GTGTCTCGGGCCTAAACACATGATAAACAAAGTCATAAGCACATGGATCACATACAGGAAATATGTATATAACATTAAAGATATAACTTTTTATT 3420
CACAGAGCCCGGATTTGTGTACTATTTGTTTCAGTATTCGTGTACCTAGTGTATGTCCTTTATACATATATTGTAATTTCTATATTGAAAAATAA
Exon 3

AAAAAAAGGGGAACACAAGTCCCGACACGTACCGTGGCACCTTGGAGGAAGGGCCCTCGTCAGGATTATCAGGGTCCATCTTTCTCTTGGCAGAG 3515
TTTTTTTCCCCTTGTGTTCAGGGCTGTGCATGGCACCGTGGAACCTCCTTCCCGGGAGCAGTCCTAATAGTCCCAGGTAGAAAGAGAACCGTCTC
Exon 2

GACTCCATCGTGTCAAGGACGGTGACTGCAGAAAGGATCCATGGAAAGGAACAGTCTGTTAGTCTGTCAGCTATTATGTCTGGTGGCGCGCGCGG 3610
CTGAGGTAGCACAGTTCCTGCCACTGACGTCTTTCCTAGGTACCTTTCCTTGTCAGACAATCAGACAGTCGATAATACAGACCACCGCGCGCGCC

Exon 2

CAGCAACGAGTACTGCTCAGACTACACTGCCCTCCACCGTTAACAGCACCGCAACAGGAGTTACCTCTGACTCTCAACAGAACACAACTCAGCTG 3705
GTCGTTGCTCATGACGAGTCTGATGTGACGGGAGGTGGCAATTGTCGTGGCGTTGTCCTCAATGGAGACTGAGAGTTGTCTTGTGTTGAGTCGAC

MIT_LM2 CTCF motif PUBLISHED

CCTGCTTCTTCTGCTGCTGCCTTAAGTCTTCCATCTGCGTCAGCGGTGCAAGCCCATTCCCCGAGCTCATTTTCAGACACATACCCTACCGCCAC 3800
GGACGAAGAAGACGACGACGGAATTCAGAAGGTAGACGCAGTCGCCACGTTCGGGTAAGGGGCTCGAGTAAAAGTCTGTGTATGGGATGGCGGTG

GGCCTTGTGCGGCACACTGGTGGTGGTGGGCATCGTGCTGTGCCTAAGTCTGGCCTCCACTGTTAGGAGCAAGGAGCTGCCGAGCGACCATGAGC 3895
CCGGAACACGCCGTGTGACCACCACCACCCGTAGCACGACACGGATTCAGACCGGAGGTGACAATCCTCGTTCCTCGACGGCTCGCTGGTACTCG

CGCTGGAGGCATGGGAGCAGGGCTCGGATGTGGAAGCTCCGCCGCTACCGGAGAAGAGCCCATGTCCGGAACACGTACCCGAGATTCGCGTGGAG 3990
GCGACCTCCGTACCCTCGTCCCGAGCCTACACCTTCGAGGCGGCGATGGCCTCTTCTCGGGTACAGGCCTTGTGCATGGGCTCTAAGCGCACCTC

ATCCCACGCTATGTTTAATAAAAACTGCGGGTACTGGGGACGGTGTTGTTGTATATGTGAATTTGTAAATAATAAATGGACCCCATCCTGTAAA 4085
TAGGGTGCGATACAAATTATTTTTGACGCCCATGACCCCTGCCACAACAACATATACACTTAAACATTTATTATTTACCTGGGGTAGGACATTT

AATACAGAGTCCGTGTCAGTCTCTGAAGGACAGAGTATTGGCATATAGCCAATAAAGAGAGTTGTGGCAAAGAGCCATGTTATGGATTAGTAATG 4180
TTATGTCTCAGGCACAGTCAGAGACTTCCTGTCTCATAACCGTATATCGGTTATTTCTCTCAACACCGTTTCTCGGTACAATACCTAATCATTAC

GAAAGTATCGCCACCAATAGAGGAGTGGTCAATAATGGTCAATAACCCACACCTATAGGCTAAGCTATACCATCACCTATAGCATAAGGAAGCGG 4275
CTTTCATAGCGGTGGTTATCTCCTCACCAGTTATTACCAGTTATTGGGTGTGGATATCCGATTCGATATGGTAGTGGATATCGTATTCCTTCGCC

GGGTGTATAGACCCCAAGCCAAAAACAGTATAGCATGCATAAGAAGCCAAGGGGGTGGGCCTATAGAGTCTATAGGCGGTACTTACGTCACTCTT 4370
CCCACATATCTGGGGTTCGGTTTTTGTCATATCGTACGTATTCTTCGGTTCCCCCACCCGGATATCTCAGATATCCGCCATGAATGCAGTGAGAA

Exon 1

GGCACGGGGAATCCGCGTTCCAATGCACCGTTCCCGGCCGCGGAGGCTGGATCGGTCCCGGTGTCTTCTATGGAGGTCAAAACAGCGTGGATGGC 4465
CCGTGCCCCTTAGGCGCAAGGTTACGTGGCAAGGGCCGGCGCCTCCGACCTAGCCAGGGCCACAGAAGATACCTCCAGTTTTGTCGCACCTACCG

Exon 1

GTCTCCAGGCGATCTGACGGTTCACTAAACGAGCTCTGCTTATATAGACCTCCCATAGTAAACGCCTACCGCCCATTTGCGTCAATGGGGCGGAG 4560
CAGAGGTCCGCTAGACTGCCAAGTGATTTGCTCGAGACGAATATATCTGGAGGGTATCATTTGCGGATGGCGGGTAAACGCAGTTACCCCGCCTC

Exon 1

transcription start site

TTATTACGACATTTTGGAAAGTCCCGTTGAATTTGGTGCCAAAACAAACTCCCATTGACGTCAATGGGGTGGAGACTTGGAAATCCCCGTGAGTC 4655
AATAATGCTGTAAAACCTTTCAGGGCAACTTAAACCACGGTTTTGTTTGAGGGTAACTGCAGTTACCCCACCTCTGAACCTTTAGGGGCACTCAG

AAACCGCTATCCACGCCCATTGATGTACTGCCAAAACCGCATCACCATGGTAATAGCGATGACTAATACGTAGATGTACTGCCAAGTAGGAAAGT 4750
TTTGGCGATAGGTGCGGGTAACTACATGACGGTTTTGGCGTAGTGGTACCATTATCGCTACTGATTATGCATCTACATGACGGTTCATCCTTTCA

CCCGTAAGGTCATGTACTGGGCATAATGCCAGGCGGGCCATTTACCGTCATTGACGTCAATAGGGGGCGTACTTGGCATATGATACACTTGATGT 4845
GGGCATTCCAGTACATGACCCGTATTACGGTCCGCCCGGTAAATGGCAGTAACTGCAGTTATCCCCCGCATGAACCGTATACTATGTGAACTACA

EMBL_M1 CTCF motif

ACTGCCAAGTGGGCAGTTTACCGTAAATACTCCACCCATTGACGTCAATGGAAAGTCCCTATTGGCGTTACTATGGGAACATACGTCATTATTGA 4940
TGACGGTTCACCCGTCAAATGGCATTTATGAGGTGGGTAACTGCAGTTACCTTTCAGGGATAACCGCAATGATACCCTTGTATGCAGTAATAACT

CGTCAATGGGCGGGGGTCGTTGGGCGGTCAGCCAGGCGGGCCATTTACCGTAAGTTATGTAACGCGGAACTCCATATATGGGCTATGAACTAATG 5035
GCAGTTACCCGCCCCCAGCAACCCGCCAGTCGGTCCGCCCGGTAAATGGCATTCAATACATTGCGCCTTGAGGTATATACCCGATACTTGATTAC

ACCCCGTAATTGATTACTATTAATAACTAGTCAATAATCAATGTCAACATGGCGGTCATATTGGACATGAGCCAATATAAATGTACATATTATGA 5130
TGGGGCATTAACTAATGATAATTATTGATCAGTTATTAGTTACAGTTGTACCGCCAGTATAACCTGTACTCGGTTATATTTACATGTATAATACT

TATAGATACAACGTATGCAATGGCCATTAGCCAATATTGATTTATGCTATATAACCAATGACTAATATGGCTAATGGCCAATATTGATTCAATGT 5225
ATATCTATGTTGCATACGTTACCGGTAATCGGTTATAACTAAATACGATATATTGGTTACTGATTATACCGATTACCGGTTATAACTAAGTTACA

ATATATATCGATATGCATTGGCCATGTGCCAACTTGGTATCGCCTCTATCGGCGATATGGCCTCATATCGTCTGTCACCTATATCGAAACTGCGA 5320
TATATATAGCTATACGTAACCGGTACACGGTTGAACCATAGCGGAGATAGCCGCTATACCGGAGTATAGCAGACAGTGGATATAGCTTTGACGCT

TATTTGCGACACACAGAATCGCCCAAGTCGCCAAAGTCGTCTATCGCCATCCCCGTAAACGATATAAGCGTTATCGCCAGATATCGCATATGCC 5415
ATAAACGCTGTGTGTCTTAGCGGGTTCAGCGGTTTCAGCAGATAGCGGTAGGGGGCATTTGCTATATTCGCAATAGCGGTCTATAGCGTATACGG

CAAAAATCACTTTTTAAAAAATGGCGATATCAGTTACACAGAAACTCACATCGGCGACATTTTCAATATGCCATATTTTCAAATATCGATTCTTC 5510
GTTTTTAGTGAAAAATTTTTTACCGCTATAGTCAATGTGTCTTTGAGTGTAGCCGCTGTAAAAGTTATACGGTATAAAAGTTTATAGCTAAGAAG

CAATATCGCCATCTCTATCGGCGATAAACACCACTATCGCGCGACATGAATTTAGTCGGCGACAGAAATCTCGAAACGCGTATTTCGGACAAACA 5605
GTTATAGCGGTAGAGATAGCCGCTATTTGTGGTGATAGCGCGCTGTACTTAAATCAGCCGCTGTCTTTAGAGCTTTGCGCATAAAGCCTGTTTGT

CACATTTTATTATTCACTGCAGCATATAGCCCATTTTAGCGCGGCACACATCCAGCCGTTTGTGTTTCTTAACGCTCTCCAGGTACTGATCCAGG 5700
GTGTAAAATAATAAGTGACGTCGTATATCGGGTAAAATCGCGCCGTGTGTAGGTCGGCAAACACAAAGAATTGCGAGAGGTCCATGACTAGGTCC

CCCACGATCCGGGTTATCTTGTCGTATTCCAGATTGATCCATCAATAGGGAACGCTGCCAGCGGCGCCCAGCAGGTACTGCGCCTTGTCGTTTAC 5795
GGGTGCTAGGCCCAATAGAACAGCATAAGGTCTAACTAGGTAGTTATCCCTTGCGACGGTCGCCGCGGGTCGTCCATGACGCGGAACAGCAAATG

TTTGCCGCAGCGTATTCGCCCGTCAGCTTCGAGGTATAACCTACAACACGGAGGGGAAGGGGGGTACAAAACGTGAAATTAGACTTTTTTTTTA 5890
AAACGGCGTCGCATAAGCGGGCAGTCGAAGCTCCATATTGGATGTTGTGCCTCCCCTTCCCCCCATGTTTTGCACTTTAATCTGAAAAAAAAAT

ATGATGTTTTGTCCCTCTCTGTCTTACTCTCCCATAGGCTGTAAGGCCCTCAGGAAGAGACTTACGGATTGTAGTTGCAGCTCGTCAGTTTGTTG 5985
TACTACAAAACAGGGAGAGACAGAATGAGAGGGTATCCGACATTCCGGGAGTCCTTCTCTGAATGCCTAACATCAACGTCGAGCAGTCAAACAAC

TGTACGACCTGGCGTGTCAATGAATGGGTCATGGTGGTGACGATCCCGCGAATCTCAGCCGTTTTCTCGGGACTGTAGCAGACTTCGCCGTCCGG 6080
ACATGCTGGACCGCACAGTTACTTACCCAGTACCACCACTGCTAGGGCGCTTAGAGTCGGCAAAAGAGCCCTGACATCGTCTGAAGCGGCAGGCC

ACACCGCAGCCTGTGGATTCATGAAAATCTACTCTGGCATTCCCGAGGATCGTCGATGGAACATGGCTATCAGAAACGTCGAGAGACAGATCCAG 6175
TGTGGCGTCGGACACCTAAGTACTTTTAGATGAGACCGTAAGGGCTCCTAGCAGCTACCTTGTACCGATAGTCTTTGCAGCTCTCTGTCTAGGTC

ACGCACCACAGAACGCAGACAATCATGAAAATACGTACGCGACGGTGAAGCGATTGCACATTTTGAAATCGTAACAGCGTTCCGGCGGGTGGTTG 6270
TGCGTGGTGTCTTGCGTCTGTTAGTACTTTTATGCATGCGCTGCCACTTCGCTAACGTGTAAAACTTTAGCATTGTCGCAAGGCCGCCCACCAAC

ACGTTTATGAATTCGCAACATTCTTCTGCGCGCACCCGCGGCACGCGGCTGTGACCCAATAGCAGCCACAACGCCGTCAAGAACGGCGTCAGATC 6365
TGCAAATACTTAAGCGTTGTAAGAAGACGCGCGTGGGCGCCGTGCGCCGACACTGGGTTATCGTCGGTGTTGCGGCAGTTCTTGCCGCAGTCTAG

TTTGGGACTCATGACGCGCGGTTTTCAAAATTCCCTGCGCGCGCGA 3′
6411
AAACCCTGAGTACTGCGCGCCAAAAGTTTTAAGGGACGCGCGCGCT 5′
